# Supplementary figures and images for: Similar sensorimotor transformations control balance during standing and walking
Source: PLoS Comput Biol. 2021 Jun 25;17(6):e1008369. doi: 10.1371/journal.pcbi.1008369 (PMC8266079; doi:10.1371/journal.pcbi.1008369)

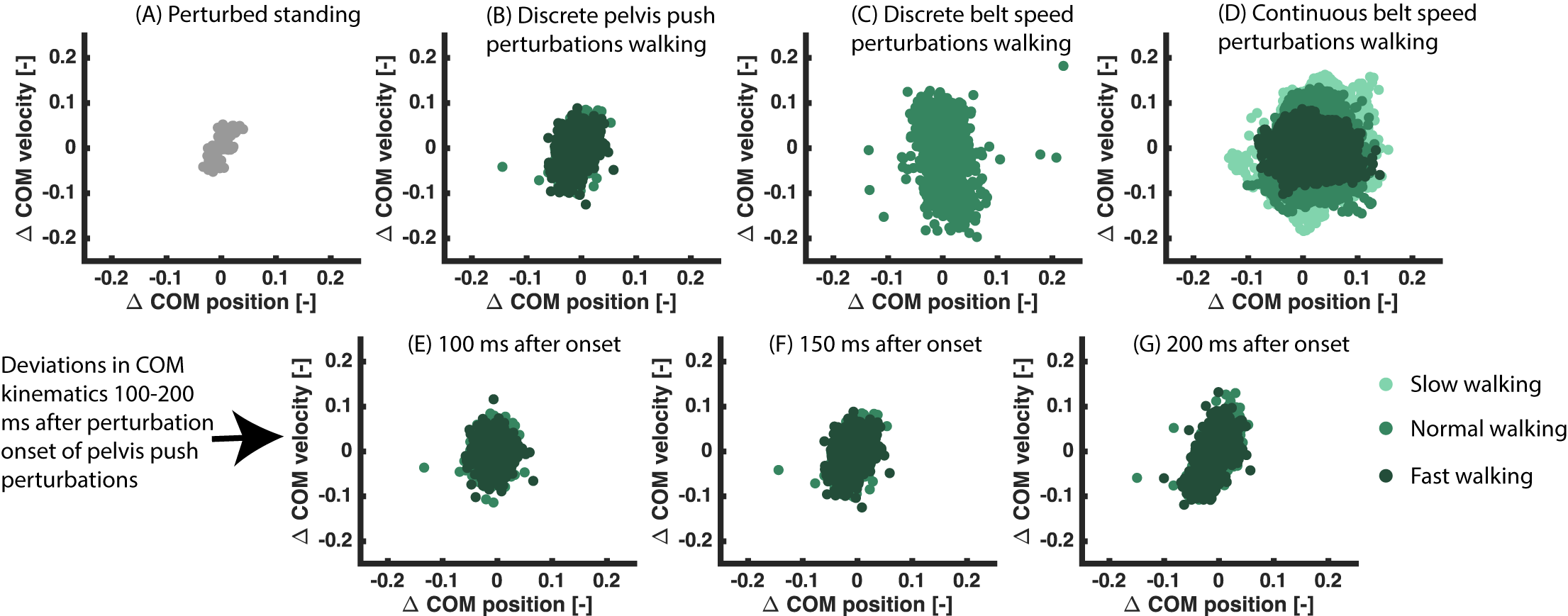

Supplement: S2 Fig — To evaluate if the magnitude of the perturbations are similar between datasets, we compared the deviation in COM position and velocity at 150ms after perturbation onset for the four datasets. We found that the deviation in COM position and velocity is similar in the different datasets of perturbed walking (B-D) and is larger in perturbed walking compared to perturbed standing (A). A similar deviation in COM kinematics is observed when analysing the data 100ms (E), 150ms (F) and 200ms (G) after perturbation onset in the pelvis push perturbations. (TIF) [file pcbi.1008369.s002.tif]
