# Supplementary material for: Similar sensorimotor transformations control balance during standing and walking
Source: PLoS Comput Biol. 2021 Jun 25;17(6):e1008369. doi: 10.1371/journal.pcbi.1008369 (PMC8266079; doi:10.1371/journal.pcbi.1008369)
Supplement: S1 Table — We evaluated whether the variance in ankle moment explained by the linear regression is sensitive to the time delay. This additional analysis was important to verify that the observed relation between COM kinematics and ankle moment is a feedback control process, and does not simply reflect the coupling due to the dynamics of the skeleton. We did this sensitivity analysis on the pelvis push perturbations and found that the relation between ankle moment and COM kinematics is indeed sensitive to the neural delay. The variance explained by the linear regression was optimal with a physiological plausible delay of 100ms and decreased strongly for delays smaller than 50 ms or larger than 120 ms. Note that time delay in this study was selected based on literature [29] and was not based on this sensitivity analysis. (PDF) [file pcbi.1008369.s003.pdf]

	Constant		Constant Fy		Constant COP		F-test		Posthoc test	
	mean	std	mean	std	mean	std	F	p	p-Fy	p-COP
Walking 0.8 m/s	0.34	0.07	0.34	0.08	0.35	0.08	1.79	0.19	x	x
Walking 1.2 m/s	0.33	0.09	0.31	0.10	0.33	0.10	4.46	0.02	0.01	0.88
Walking 1.6 m/s	0.14	0.09	0.13	0.09	0.13	0.08	0.98	0.39	x	x

Table 1: **Sensitivity of the time delay:** We evaluated whether the variance in ankle moment explained by the linear regression is sensitive to the time delay. This additional analysis was important to verify that the observed relation between COM kinematics and ankle moment is a feedback control process, and does not simply reflect the coupling due to the dynamics of the skeleton. We did this sensitivity analysis on the pelvis push perturbations and found that the relation between ankle moment and COM kinematics is indeed sensitive to the neural delay. The variance explained by the linear regression was optimal with a physiological plausible delay of 100ms and decreased strongly for delays smaller than 50 ms or larger than 120 ms. Note that time delay in this study was selected based on literature [**?**] and was not based on this sensitivity analysis.
