# Supplementary material for: Similar sensorimotor transformations control balance during standing and walking
Source: PLoS Comput Biol. 2021 Jun 25;17(6):e1008369. doi: 10.1371/journal.pcbi.1008369 (PMC8266079; doi:10.1371/journal.pcbi.1008369)
Supplement: Attachment — Submitted filename: ResponseToReviewers.pdf [file pcbi.1008369.s011.pdf]

The questions from the reviewers are in blue, the answers to the questions are shown in **black** and the adjustments to the manuscript in **green.** Please note that we also provided a pdf document with tracked changes to facilitate reviewing our adjustments in the manuscript.

Reviewer 1:

Major comment: One of the things I'm kind of left confused about is the role of simple stretch reflexes in all of this; we know they exist (see all the work on H-reflexes that the authors also mention), and that they should have a more or less 1-to-1 mapping between ankle state and muscle activity. Yet, the data of the authors shows that these models fit less well, which, in a way, is surprising to me. Probably, this is due to the fact that the models that are fitted here do not only contain information on short-latency reflexive activity, but also on longer latency mechanisms. Or is there something else that I am missing here? These kind of thoughts could be expressed somewhat more clear in the manuscript.

This is indeed an important remark and we agree that this should be discussed in more detail in the manuscript. As the reviewer suggests, the bad fit of the local reflex model can be explained by the time instant at which we evaluated the relation between EMG/torques and COM kinematics. From about 50 to 100ms after the perturbation onset, changes in electromyography(EMG) and joint moments are expected to be the combined result of both short-latency reflexes and longer latency mechanisms (supraspinal feedback might also contribute). We currently selected the input for the regression model (ankle angle and COM deviations) 150ms after perturbation onset. For the EMG activity, we used 190ms given the neural delay of 40ms in the local feedback model. This decision of 150ms delay between perturbation onset and the evaluation of the feedback model response is based on the observation that large changes in muscle activity and joint moments were observed at this time instance for all different types of perturbations.

I added a new paragraph in the discussion section to clarify this to the reader:

The observation that reactive muscle activity and joint moments were better described by a model with delayed feedback of COM kinematics, compared to delayed feedback of ankle kinematics, does not mean that local reflexes are not important for human balance control. We only showed that our model, based on linear feedback from ankle angle and velocity cannot explain the reactive muscle activity and joint moments respectively 190ms and 230ms after perturbation onset. The reason for this may be twofold: First, alternative local feedback models, for example based on feedback from muscle fiber kinematics or force, might improve the variance explained by the local feedback model. Second, 200ms after perturbation onset, the reactive muscle activity observed in the EMG signals and joint moments could be the result from both local reflexes and long latency reflexes in response to the ongoing perturbations. The high variance explained by the COM kinematics feedback model indeed suggests that the response, at the time instance we evaluated it, does not merely result from local reflexes. Hence, our results indicate that, similar as in standing balance [2], long latency responses reflect delayed feedback of task-level variables and not linear feedback of joint kinematics errors.

Minor comments:
1) Figure 2; what are the green lines in this (and other) figures? It seems as if they are fits to just the unperturbed data for a, but for b, it seems for all data (or is that due to the multiple regression that was performed? Also, I was wondering why for the standing, only the COM feedback model is shown, and not the data for the ankle feedback model?

The green lines represent the coefficients (K_p and K_v) of the multiple regression: $\Delta T_a(t) = K_p\, \Delta COM(t-\tau) + K_v C\dot{O}M(t-\tau)$). We decided to include this in figure 2 to make this figure consistent with the analysis of perturbed walking were we discuss the change in the coefficients during the gait cycle (i.e. figure 6 and 7).

I adapted the caption in the figure: "Least squares regression was used to estimate the position (Kp) and velocity (Kv) feedback gains (i.e. slope of the green lines in A and B).

I added the data of the ankle feedback model as well (Note that reviewer 2 asked a similar question).

2) Figure 3; from the numbers, the RMSE in G is twice that of D, but if I would have to guess, I would say it's much larger? (or is this just my eyes being off?

The reviewer is indeed correct. There was a mistake in the computation of the RMSE. In the original manuscript, we included the unperturbed data in the analysis of the model with one set of gains for forward and backward directed perturbations. This had quite a large influence on the RMSE as 53% of all data points are unperturbed gait cycles. The variance in the unperturbed gait cycles is low, and as a result the error in the reconstructed moment is also relatively low. Hence, the RMSE over all data points is lower than the RMSE over the data points from perturbed gait cycles only. We therefore adapted table 2 and the values in Figure 3 in the new manuscript.

I updates the values in Figure 3 and the results in table 2.

3) It may be nice to show some example traces for one perturbation of COM deviations, and then ankle moment deviations, and estimated ankle moment deviations, just for the reader to get some idea of what's going on.

I added a new figure to the manuscript to explain the relation between the COM position, velocity and ankle moment as a function of time, and how we performed a least-square regression on this data to evaluate the feedback model (Figure 2 in the new manuscript). I hope that this figure clarifies (a) the perturbations, (b) the regression method, and (c) how we selected the discrete time points to evaluate the least-squares regression.

4) Figure 4. In figures 2-3 you used arrows with arrowheads pointing toward the feature, and at the tail the thing it indicated. Here, you do the opposite (super-minor, but may be nice to be consistent in this).

I updated figure 4.

5) Page 6, line 183; here, it is stated that the perturbations happened around toe-off, yet the figure that is refereed to has perturbations in many phases of the gait cycle?

This was indeed unclear. In this paragraph, we want to explain that the fit of the COM-feedback model was similar in the belt-speed perturbations and in the pelvis-push perturbations in case the perturbation was applied at the same instance of the gait cycle. Since the pelvis-push perturbation were (only) applied around toe-off, we only describe belt speed perturbations around 20% of the stride in the belt speed perturbations (Figure 5). The results of the belt-speed perturbations, applied during different phases in the gait cycle, are discussed in the next section (i.e. section 2.4). We

adapted the manuscript (see below) and removed the reference to figure 5 in this paragraph since this does not contain additional information for this paragraph.

Second, we performed the same analysis in a dataset with a sudden increase or decrease in speed of the treadmill belts. To compare both perturbations modalities, we only considered belt-speed perturbations that were applied at the same time instant of the gait cycle as the pelvis push perturbations (i.e. around toe-off of the contra lateral leg). Similar as in the pelvis push perturbations, we found that the reactive ankle moment after the perturbation could be largely reconstructed by delayed COM feedback (R^2= 0.61).

6) Figures 6 and 7; I think the figures miss a delta in both the x and y labels, as these are deviations from the nominal (pattern/ activity/etc), right?

This is not the case in figure 6 and 7. We chose to visualize the total joint moments (and muscle activity) in this figure instead of the deviation from average moments in each gait phase. We believe that this improves the clarity of the figure since this also clearly shows the (average) change in joint moment and muscle activity during the unperturbed gait cycle. This is especially important for the subplots with the muscle activity. Since we include muscle activity of unperturbed gait cycles in this figure, you can clearly see the inhibition (i.e. reduction) of muscle activity after a perturbation when the muscle is active in the specific gait phase (I believe the best example is the Soleus in figure 6).

We adapted the caption of figure 6 and 7 to inform the reader about this. "Note that the total joint moment and muscle activity rather than the deviation from the average unperturbed data is shown to provide information on the muscle activity and joint moments during unperturbed walking.

7) Page 9, line 277, one of our recent preprints may be of interest here; basically, we showed phase dependent contributions of vestibular info, which also was context dependent. See https://www.biorxiv.org/content/10.1101/2020.09.30.319434v1

Thank you for this reference, this is indeed a very relevant paper. We added this reference in the discussion section.

8) Page 10, line 234; references that subjects might rely more on stepping (at least in the ML plane) are:
Stimpson, K.H., et al., Effects of walking speed on the step-by-step control of step width. J Biomech, 2018. 68: p. 78-83.
And
https://www.biorxiv.org/content/10.1101/2020.06.10.143867v1

Thank you for the highly relevant reference. We added this citation.

9) Methods; R^2 values are typically not normally distributed, and some sort of a (modified) fisher r-to-z transform is needed before averaging them, or doing stats on them.

We would like to thank the reviewer for this comment. We used the Fisher Z-Transformation to transform the R values so that they become normally distributed and performed a t-test on the normally disturbed data.

We adapted the section: *"methods-statistical comparison feedback models"* in the manuscript.

"**A Fisher z-transformation was used to transform the correlation coefficient in normally distributed z-scores.** Bonferroni correction was used to correct for multiple comparisons in the post-hoc testing. To test the second hypothesis, we also used a paired t-test**, with R-values after Fisher z-transformation,** to evaluate if the R value is significantly different in the model with task-level feedback compared to a model with joint-level feedback. A two-sided confidence interval with an alpha level of 0.05 was used for all statistical tests.

Reviewer #2:

Major general comments:

The manuscript lacks the nuanced presentation that, in my opinion, is necessary for such modeling studies. For instance, it is stated in the abstract that "We found that delayed feedback of center of mass position and velocity, but not local feedback of joint positions and velocities, can explain…" and in the results section that "… we showed that … can largely be explained by task-level feedback of COM kinematics and not with local feedback of joint kinematics" However, the study does not show that ankle responses cannot be explained by local feedback; it shows that the local feedback model the authors selected cannot, and it is still possible that a different local feedback model could explain the ankle responses observed in humans. I think this is a critical limitation of the current manuscript and suggest the following modifications:

1) Do not say "local feedback cannot …" as your conclusion. Instead, say "the local feedback model cannot …"

This is indeed an important remark. We adapted the results and discussion sections of the manuscript in several places to account for this remark. You can find all the adjustments in the document with track changes.

2) In the introduction, discuss the nature of modeling studies. Again, showing that a local feedback model you selected cannot fit/correlate/etc. observed data does not show that local feedback cannot explain observed data.

We believe that this is mainly a discussion point and therefore adapted the discussion section to account for this important remark. In the section related to the model that (might) represent tactile feedback, we added the observation that our modelling assumptions might be invalid.

First, this might indicate that tactile information in the foot cannot explain the modulation of COM feedback gains during the gait cycle. **Second, this might also indicate that the simplifying assumption of modelling information from cutaneous sensors with linear feedback of vertical ground reaction force or COP location is not valid.** In the future, cutaneous stimulations experiments could generate more insight in the modulation of task-level feedback gains during walking.

In addition, in the new section related to task-level versus joint-level feedback, we added a discussion about our modelling assumptions related to joint-level versus task-level feedback.

The observation that reactive muscle activity and joint moments were better described by a model with delayed feedback of COM kinematics, compared to delayed feedback of ankle kinematics, does not mean that local reflexes are not important for human balance control. We only showed that our model, based on linear feedback from ankle angle and velocity cannot explain the reactive muscle activity and joint moments respectively 190ms and 230ms after perturbation onset. The reason for this may be twofold: First, alternative local feedback models, for example based on feedback from muscle fiber kinematics or force, might improve the variance explained by the local feedback model. Second, 200ms after perturbation onset, the reactive muscle activity observed in the EMG signals and joint moments could be the result from both local reflexes and long latency reflexes in response to the ongoing perturbations. The high variance explained by the COM kinematics feedback model

indeed suggests that the response, at the time instance we evaluated it, does not merely result from local reflexes. Hence, our results indicate that, similar as in standing balance [2], long latency responses reflect delayed feedback of task-level variables and not linear feedback of joint kinematics errors.

3) You could/should introduce and discuss that there can be and there are other (task-based and) local models than the ones you selected. For instance, [Song & Geyer, A neural circuitry that emphasizes spinal feedback generates diverse behaviours of human locomotion. The Journal of physiology, 2015] shows that local force feedback for ankle control can contribute to producing walking and maintaining balance. While the model includes a foot placement control (as you cited as "balance is mainly controlled by foot placement […,33]") it also has local ankle control.

We adjusted the introduction and discussion section to clarify that there are other feedback models than the ones we selected (see also answer to comments above).

In the introduction we added: In contrast with models for standing balance, the control of ankle muscle activity in neuromechanical models of walking, is modelled using local feedback (e.g., delayed force feedback) or foot placement rather than task-level feedback from COM kinematics \cite{Song2017}.

We also adapted the discussion section: **"Derive neuromechanical models for balance control during walking"**

Our findings have important implications for modelling neuromechanics of walking. In current models, balance is mainly controlled by foot placement \cite{Song2017, Wang2014a}. Here, we showed that subjects also adjust the ankle joint moment in response to perturbation during walking and that this strategy can be modelled using delayed COM position and velocity feedback. Hence, we propose an additional supra-spinal feedback loop to model the ankle strategy during walking, similar to the neuromechanical models of perturbed standing \cite{Welch2008}. Since COM kinematics feedback largely captures reactive ankle joint moment in response to different types of balance perturbations across walking speeds, we believe that this is an important extension to current state-of-the art neuromechanical models. One of the future challenges is to investigate how this additional COM-feedback loop interacts with local feedback control, for example positive force feedback of the calf muscles as modeled by \cite{Geyer2006}. We believe that a combination of local and supra-spinal feedback to control the ankle muscles is especially useful in the control of wearable robotic devices. We hypothesize that an additional supra-spinal feedback loop will facilitate shared control of balance when using neuromechanical models in the control of wearable robotic devices.

(Note, this paragraph was also edited based on your question 5-1).

4) In the introduction, you should also justify the models you selected/proposed in your study. I think the linear models you selected are justifiable as it is simple to be considered as a baseline model and have been used in previous studies on standing balance. (In that, if the models have been used in or have been adapted from previous studies, you should clearly state and cite them in the introduction and also when presenting eq (1)~(4).

I edited the first and last paragraph of the introduction to make it more explicit that the COM feedback model has been used in previous studies on standing balance **(see sentences in bold).**

Experiments involving external mechanical perturbations of standing have found evidence of task-level feedback from COM kinematics. Delayed feedback from COM kinematics can explain reactive muscle activity and changes in joint moments in response to perturbations during standing \cite{Ting2009,Lockhart2007,Welch2009}. **Here we used an identical feedback model to investigate if**, similar as in standing balance, COM position and velocity feedback can describe sensorimotor transformations during walking.

In this study, we investigate the sensorimotor transformations underlying the ankle strategy to control balance during walking. We hypothesize that, comparable to standing, feedback from COM kinematics, and not local joint-level feedback, can explain reactive ankle muscle activity and ankle joint moments in perturbed walking. However, given the changes in the stability of the musculoskeletal system and changes in body kinematics during the gait cycle and with walking speed, we hypothesize that feedback gains will be modulated during the gait cycle and with gait speed based on input from tactile sensors. To test these hypotheses, we evaluated the relation between the inputs of the feedback (delayed COM and ankle joint kinematics) and the resulting motor command (muscle activity and joint torques) in four available datasets of perturbed standing and walking at different walking speeds in young healthy adults. **This linear feedback model is based on previous studies on standing balance [ref] and was adapted to allow modulation of feedback gains during the gait cycle.** We used different perturbation modalities, support surface translations and pelvis pushes, to excite the (neuromechanical) system in multiple ways (Fig. \ref{fig:Datsets}), thereby guaranteeing the generalizability of our findings.

5) The discussion can be stronger by covering relevant musculoskeletal modeling studies as (there are not too many and) can be suitable for more nuanced discussions:
5-1) Section 3.2 can include the discussion of “the changes in muscle responses do not necessarily indicate modulation of reflex gains” as stated in [33], as the changes in the responses could result from the musculoskeletal configuration/dynamics. I think this is worth emphasizing to point out the limitation of your studies using simple linear models that do not incorporate the complex musculoskeletal dynamics.

Indeed, changes in muscle responses to a perturbation throughout the gait cycle do not necessarily indicate modulation of reflex gains. Theoretically, it might be possible to find a control architecture that can explain the changes in muscle response without gain modulation. Note that we tested a hypothesis: (delayed linear) feedback from COP position and vertical ground reaction forces improves the fit of a constant gain model. We could not confirm this hypothesis. Obviously, this does not disprove that information of cutaneous sensors is used to modulate feedback gains. We only showed that feedback architectures based on delayed feedback of COM kinematics or delayed feedback of ankle kinematics, with constant gains or with feedback gains that were linearly dependent on the COP location or vertical ground reaction force, cannot explain the changes in muscle responses throughout the gait cycle (figure 9, B).

We dit not model the complex muscular dynamics, but we did model the skeleton dynamics. Note that we used a data-driven or inverse approach where we estimated joint kinematics and joint moments, from the recorded ground reaction forces and marker coordinates using inverse kinematics/dynamics procedures. This information was combined with electromyography measurement. Indeed, without modelling the muscular system, this approach is limited to evaluating simple feedback laws based on the states that we can measure (joint angles, velocities) and not the state of the muscles for example. We adapted the discussion section (Task-level versus joint-level feedback to account for this remark.

The observation that reactive muscle activity and joint moments were better described by a model with delayed feedback of COM kinematics, compared to delayed feedback of ankle kinematics, does not mean that local reflexes are not important for human balance control. We only showed that our model, based on linear feedback from ankle angle and velocity cannot explain the reactive muscle activity and joint moments respectively 190ms and 230ms after perturbation onset. The reason for this may be twofold: First, alternative local feedback models, for example based on feedback from muscle fiber kinematics or force, might improve the variance explained by the local feedback model. Second, 200ms after perturbation onset, the reactive muscle activity observed in the EMG signals and joint moments could be the result from both local reflexes and long latency reflexes in response to the ongoing perturbations. The high variance explained by the COM kinematics feedback model indeed suggests that the response, at the time instance we evaluated it, does not merely result from local reflexes. Hence, our results indicate that, similar as in standing balance [2], long latency responses reflect delayed feedback of task-level variables and not linear feedback of joint kinematics errors.

(Note, this is the same paragraph as in your question 3).

5-2) Section 3.4 can mention the potential contribution of feedforward control in balancing as suggested in [Haeufle, Schmortte, Geyer, Müller & Schmitt, The benefit of combining neuronal feedback and feed-forward control for robustness in step down perturbations of simulated human walking depends on the muscle function. Frontiers in computational neuroscience, 2018].

We investigated the reactive response to the perturbation since the perturbation were applied at variable time instance. We believe that feedforward control can contribute in two ways to the balance recovery response to the unexpected perturbation.

(1) The subjects might modulate feedback gains in anticipation of a perturbation with unknown timing, direction and magnitude
(2) The subjects might change baseline muscle activity during normal walking to adjust the intrinsic component of joint impedance (i.e. joint stiffness due to visco-elastic behavior of muscle fibers).

I believe that we already discuss both feedforward control strategies in our manuscript.

1. First, we identified the gains based on perturbation data. Therefore, the identified gains include the possible feedforward adaptions in feedback gains (i.e. point 1).
2. We investigated the relation between COM kinematics and reactive muscle activity both at the level of joint moments and reactive muscle activity. If the changes in ankle joint moments could be solely attributed to the visco-elastic properties of muscle fibers (and not reactive muscle activity), one would observe this relation only at joint level and not at the level of muscle activity.

We believe that we discussed this in the section relation to **contribution of reactive muscle activity**.

- The presentation is not in its best form. Please find my comments below for more details.

Detailed Comments:

Line 52~53: It is written that the ankle strategy is the “dominant” strategy in the sagittal balance control. However, the following content does not seem enough to support that statement. Either rephrase the sentence, elaborate on what you mean by “dominant”, or add evidence that the ankle strategy is dominant over the stepping strategy.

With this statement, we wanted to highlight that the ankle strategy has an important contribution in in sagittal balance control. This statement mainly relies on studies that showed that:

1) The adjustment of foot placement is very small in response to sagittal plane perturbations, which indicates that another strategy is used to control balance [3].
2) When the ankle strategy is blocked experimentally, a strong increase in foot placement is observed [4]. This indicates that the ankle strategy seems to be very important in the sagittal plane during walking, since the stepping strategy is used as an alternative strategy when the ankle mechanisms is blocked.

We removed the statement that “the ankle strategy is the dominant strategy” from the manuscript. We still explain the two studies mentioned above, which enables the reader to judge the relative importance of the ankle and stepping mechanisms in the sagittal plane. Hence the adapted paragraph in the introduction.

Following sagittal plane perturbations, both stepping and ankle strategies are used to control balance ~~with the latter being the dominant strategy~~. COM kinematics feedback can explain fore-after foot placement \cite{Wang2014a}. However, the correlation between COM kinematics and foot placement is weaker in the sagittal than in the frontal plane \cite{Joshi2019}, which can be attributed to the higher reliance on the ankle strategy. Indeed, when eliminating the ankle strategy through stilt walking, a strong correlation between COM kinematics and foot placement is observed in the sagittal plane \cite{Vlutters2018b}.

Lines 89~91: “We therefore hypothesize that tactile sensors also modulate task-level feedback gains and thereby contribute to the observed modulation of reactive muscle activity.” -> Consider rephrasing the sentence so that this hypothesis is secondary to your main hypothesis of task-level vs. local feedback.

I adapted the paragraph in the introduction related to this secondary hypothesis.

If COM kinematics feedback is indeed modulated during the gait cycle, this raises the question which sensory mechanisms underlie this modulation. Cutaneous stimulation studies suggest that sensory information from tactile sensors in the foot modulates reflex gains during walking \cite{Nakajima2016,Zehr2014}. We therefore tested the secondary hypothesis that tactile sensors also modulate task-level feedback gains and thereby contribute to the observed modulation of reactive muscle activity.

Lines 136~137: “Inputs (COM kinematics or ankle kinematics) and outputs (ankle moment) …” -> I suggest you rephrase the sentence so that it holds the main points without the parts in parentheses. In other words, “ankle moment” should not be in parentheses.

Inputs, i.e., COM kinematics or ankle kinematics, and outputs, i.e., **ankle moments,** were selected respectively at 150ms and 230-250ms after perturbation onset since ….

The order of the experiments in Fig. 1, 2 Results, and 4.1 Experimental methods are not consistent, thus confusing.

We changed the order in the figure and in the methods section. The order is determined by the order in the results section and therefore we adapted it as follows:

1. Standing perturbations
2. Pelvis push perturbations
3. Discrete belt speed perturbation
4. Continuous belt speed perturbations

(Adapted in Figure 1 and in the methods section).

Fig. 1, Continuous translation walking: Is the y-axis treadmill velocity or Com velocity? Please clarify.

This is treadmill velocity. This was added to the y-label in the figures.

Lines 155~156: "reactive ankle joint moments and muscle activity …" -> "reactive ankle joint moments and muscle activity during standing…"

This was adjusted in the manuscript

Please explain what you mean by "limitations related to normalizing EMG data" in section 4.3.

We explained the limitations related to normalizing EMG data (i.e. to compare EMG data between subjects) (**in Bold**).

Joint kinematics, kinetics and muscle activity data were normalized to facilitate comparison between subjects and datasets to enable estimation of feedback gains based on data pooled over all subjects. Similar as in \cite{Seethapathi2019}, COM positions were normalized by l_{max}, speeds by $\sqrt{gl_{max}}$, torques by mgl_{max} and muscle activity by MVC values for the standing data and by the peak values of the **gait-cycle-average activity observed during unperturbed walking for the walking data**. **We noticed that normalizing the EMG data was not sufficient to have a reliably comparison of muscle activity between subjects, which might be related to the lack of MVC data in the walking experiments [5]. We therefore fitted the feedback model only on EMG data of individual subjects, and not on the data pooled over all subjects.**

Fig 3. Subplot labels are missing A) and are not consistent with the caption.

I added A) to this figure and updated the caption accordingly.

I would recommend presenting Fig. 2~4 in a consistent manner. For instance, include joint feedback data in Fig 2, and combine Fig 3 and 4. Fig 2 and the combined Fig3+4 could have the same layout.

I adapted figure 2-3-4. I added the joint level feedback to figure 2 and combined figure 3 and 4 in one figure.

Table 2. RMS of TA is smaller for the Joint feedback model. p-RMS of 0.004 means that the Joint feedback model is significantly better? Please clarify.

We thank the reviewer for pointing us to mistakes in the table arising when converting the table to latex. The correct table is now added. As you can see, now both the RMS value is significantly lower for the COM feedback model compared to the local feedback model.

Note that reviewer 1 (Minor comment 2) had also comments related to this table since the reported RMS values did not match with the data points in figure 3.

Line 182~186: Is this paragraph only talking about the data around 60 %stride in Fig 5? If so, please clarify it by, for example, marking the 60 % stride data with shaded boxes and referring it to it as "(shaded area in Fig. 5)". If I am misunderstanding this paragraph, then please clarify what you mean by "(also applied around toe-off of the contralateral leg)" at line 183.

This is about 10-20% of the gait cycle (since the perturbations were applied around toe-off **of the contra-lateral leg**). This was adjusted in the manuscript (see also comment 5 of reviewer 1).

Fig. 5: Are the data for one speed?

Yes, only one speed in this dataset. I added this to the caption of figure 5 (similar as in figure 3-4).

Lines 195~196: Indicate what gait phases you are indicating to by initial, mid-, and terminal stance. E.g. terminal stance: 62.5%, etc.

This was added to the manuscript.

Fig 6, Row 2~4: Why are the regressions done together for forward and background perturbations? Or, why are they done separately for data in Fig 2 and 4?

The regression on joint moments is done together for forward and backward directed perturbations. We found that separate regression coefficients were needed at muscle level, but not at joint level, to explain the response to the perturbation. In Figure 2 (and new figure 3) you can clearly see that direction dependent gains are needed at muscle level to explain the data, but not at joint level (moment) level.

This was not explained in the methods section of the manuscript. We adapted the manuscript as follows:

To evaluate the relation between inputs (COM or ankle kinematics) and the ankle joint moment, we performed the linear regression independent of the perturbation direction (i.e. one single regression for both perturbation directions). This decision was mainly based on the observation that the variance explained by a single regression model was similar to the variance explained by separate regression models for each perturbation direction (Figure 2-3). In contrast, we found that separate feedback gains for the forward and backward directed perturbations were needed to explain the reactive muscle activity (Figure 2 H-J and Figure 3 H-J).

Fig 6&7: Are the x-axes COM or Del_COM? If they are COM please explain.

The x-axes are COM in figure 6 and 7. We chose to visualize the total joint moments (and muscle activity) in this figure instead of the deviation from average moments in each gait phase. We believe that this improves the clarity of the figure since this also clearly shows the change in joint moment and muscle activity during the gait cycle. This is especially important for the subplots the muscle activity. Since we add the baseline activity in this figure, you can clearly see the inhibition (i.e. reduction) of muscle activity after a perturbation only when the muscle is active in the specific gait phase (I believe the best example is the Soleus in figure 6). (Note: reviewer 1 asked this question as well in minor comment 6).

We adapted the caption of figure 6 and 7 to inform the reader about this. "Note that the total joint moment and muscle activity rather than the deviation from the average unperturbed data is shown here to also provide information on the muscle activity and joint moments during unperturbed walking".

Why is there no Table that summarizes the R^2 and RMS values for the continuous speed perturbation experiment?

The R^2 vand RMS values are summarized in figure 9. Given the large number of R^2 and RMS values (i.e. for multiple phases in the gait cycle), we believe that a figure is easier for the reader.

Line 242: "(Fig. 9)" -> "(Fig. 9-F)"
Line 245: "(Fig. 9)" -> "(Fig. 9-B and F)" or remove
Line 259: "(Fig. 9)" -> "(Fig. 9-C, D, G, and H)"

I adapted these references to the figures in the text.

Please check the writing/editorial mistakes throughout the manuscript. A few examples are:
- Line 39: "are less well studied" -> "are less studied" or "has received less …"
- Line 54: "fore-after" -> "fore-aft"
- Line 59: "Is is" -> "It is"
- Lines 201~202: "(one representative subject: figure 6A, all subjects figure: 5)" -> "(Fig. 5: all subjects, Fig 6: one representative subject)"
- Fig. 6, Row 1: Mark another value in the y-axis to show the scale.
- Fig. 7: Indicate % stance/stride as in Fig 6.
- Fig. 9: Place the subfigure labels and y-axis labels (e.g., D and H) to not invade the other subfigures.
- Line 326: "pcontact" -> "contact"
- "Fig 11." -> "Fig. S1" "Fig 12." -> "Fig. S12"

We adapted these (and some other) editorial mistakes. The error related to the figures in the appendix is caused by the latex template of the journal. This will be adjusted in the final edit by the journal.

Bibliography

[1] S. A. Safavynia and L. H. Ting, "Long-latency muscle activity reflects continuous, delayed sensorimotor feedback of task-level and not joint-level error," *J. Neurophysiol.*, vol. 110, no. 6, pp. 1278–1290, Sep. 2013.

[2] S. A. Safavynia and L. H. Ting, "Long-latency muscle activity reflects continuous, delayed sensorimotor feedback of task-level and not joint-level error," *J. Neurophysiol.*, vol. 110, no. 6, pp. 1278–1290, 2013.

[3] M. Vlutters, E. H. F. van Asseldonk, and H. van der Kooij, “Center of mass velocity-based predictions in balance recovery following pelvis perturbations during human walking,” *J. Exp. Biol.*, vol. 219, no. 10, pp. 1514–1523, Mar. 2016.

[4] M. Vlutters, E. H. F. van Asseldonk, and H. van der Kooij, “Reduced center of pressure modulation elicits foot placement adjustments, but no additional trunk motion during anteroposterior-perturbed walking,” *Journal of Biomechanics*, vol. 68, Elsevier, pp. 93–98, 08-Feb-2018.

[5] M. Besomi *et al.*, “Consensus for experimental design in electromyography (CEDE) project: Amplitude normalization matrix,” *J. Electromyogr. Kinesiol.*, vol. 53, p. 102438, Aug. 2020.
