# Supplementary material for: Similar sensorimotor transformations control balance during standing and walking
Source: PLoS Comput Biol. 2021 Jun 25;17(6):e1008369. doi: 10.1371/journal.pcbi.1008369 (PMC8266079; doi:10.1371/journal.pcbi.1008369)
Supplement: Attachment — Submitted filename: AnswerToReviewers.pdf [file pcbi.1008369.s012.pdf]

Reviewer 2 had some important comments on the introduction of the paper. We modified the introduction section with the aim to improve the clarity and readability. You can find the most important changes in this document (and all changes in TrackChanges.pdf).

Reviewer 2

Lines 30~31: “When standing balance is perturbed, the central nervous system estimates the movement of the whole body center of mass to activate muscles and control balance” This is too assertive in my opinion. Rather suggest strong enough citations or modify to something like “Previous studies suggest that …”

We changed this sentence to: *When standing balance is perturbed, previous studies suggest that the central nervous system estimates the movement of the whole body center of mass to activate muscles and control balance.*

It was very difficult to read the introduction. I needed to stop a lot to understand how some parts make sense in the broader context. I think a major revision of the entire section is necessary, so it is not so easy to suggest specific small changes. Thus, rather than giving specific suggestions, I will list a few specific points I think made the section difficult to read:

- Regarding the overall structure of the introduction, currently, many paragraphs consist of both the background and the specific ideas of this study (e.g., Lines 53~54, Lines 87~90, Line96~98, Lines 110~112). I think jumping back and forth between background and what is done in this study harms the readability. It could be easier if the introduction first covers the relevant background then describe what and how this study would address the questions. Maybe this can be done by rephrasing some sentences. For instance, instead of writing “… we hypothesize …” (Line 96) in the middle of a background paragraph, it could be stated as a general idea (and later in the “In this study, …” paragraph you can pick up on that idea).

We adapted the introduction section such that the first paragraph contains only information about the background of the study. In the new version, we introduce the hypotheses of the study in the last paragraph of the introduction. The document with tracked changes (TrackChanges.pdf) contains a good overview of the changes we made in the introduction section.

- Lines 41~42: “seems to be simple” and “This is remarkable…” do not read naturally together, although I understand what it means. Maybe you can change the first sentence to something like “Humans can stand and walk … without difficulty …”

We changed this part of the introduction to: *Most humans are extremely good at standing and walking without falling even in uncertain environments. This is remarkable, given the instability of the human skeletal system. Continuous adaptations of muscle activity are needed to control the relatively high position of the center of mass (COM) above a small base of support.*

- Lines 83~85: The sentence does not flow well in the paragraph. You should provide some flow/reasoning why you are making this statement here.

We agree with the reviewer, this sentence does not flow well in the paragraph and we believe that this information is not needed in the introduction section. We therefore removed this from sentence from the introduction section.

- Line 126~128: I think you should merge this paragraph with the previous one (with a good flow rather than simply connecting them).

We merged these paragraphs.
